# Epidemiological profile and follow-up of patients with pulmonary disease by non-tuberculous mycobacteria in Baixada Santista area, São Paulo

**DOI:** 10.31744/einstein_journal/2022AO5510

**Published:** 2022-02-08

**Authors:** Andréa Gobetti Vieira Coelho, Ana Carolina Chiou, Heloisa da Silveira Paro Pedro, Susilene Maria Tonelli Nardi, Erica Chimara

**Affiliations:** 1 Instituto Adolfo Lutz Santos SP Brazil Instituto Adolfo Lutz, Santos, SP, Brazil.

**Keywords:** Lung diseases/diagnosis, Lung diseases/epidemiology, Nontuberculous mycobacteria, Opportunistic infections

## Abstract

**Objective:**

To present the frequency and species diversity of non-tuberculous mycobacteria, estimate the prevalence of non-tuberculous mycobacterial pulmonary disease, describe the epidemiological profile, and determine the follow-up of patients with non-tuberculous mycobacterial pulmonary disease living in a region with a high burden of tuberculosis.

**Methods:**

This a retrospective cohort observational study using data records obtained from the *Instituto Adolfo Lutz* - Santos and from the São Paulo *Sistema de Vigilância de Tuberculose do Estado de São Paulo* in the period between 2000 and 2009. The studied variables were: socio-demographic characteristics, current and past history of tuberculosis, aspects related to diagnosis, and treatment and associated diseases.

**Results:**

We included 319 non-tuberculous mycobacteria isolates in the study, corresponding to 257 patients. The species *Mycobacterium kansasii* (28.5%) and *Mycobacterium fortuitum* (16.6%) presented the higher occurrence. In 10.9% (24) of the patients, there was a criterion for confirming a case of pulmonary disease due to non-tuberculous mycobacteria. In relation to gender and age, male and individuals over 50 years old were the most frequent. Considering the confirmed cases, 47.8% had a past history of tuberculosis.

**Conclusion:**

The lack of information about the cases is evident, since pulmonary disease due to non-tuberculous mycobacteria is not mandatory. The therapeutic regimen according to the identified species is fundamental for success in combating the infections caused by non-tuberculous mycobacteria. Besides that, information about the regional epidemiology of pulmonary disease caused by non-tuberculous mycobacteria and the search for associations with other comorbidities are important to establish the correct treatment. In order to improve surveillance of pulmonary diseases by non-tuberculous mycobacteria, we suggest the implantation of a sentinel surveillance and of population-based studies.

## INTRODUCTION

Non-tuberculous mycobacteria (NTM) are ubiquitous pathogens with wide distribution in the environment. Some of them are part of the microbiota of higher organisms, and others have been isolated as contaminants of nonsterile biological specimens.^(1)^

They are opportunistic agents, since atypical infections caused by these agents have been identified in patients with acquired immunodeficiency syndrome (HIV/AIDS).^(2,3)^ Despite the high pathogenicity in this population, infection caused by these microorganisms is not considered a public health problem, and, therefore, their reporting is not mandatory,^(4)^ with the exception of fast-growing mycobacteria that cause infections after invasive procedures.^(5)^

The published literature describes aerosol dispersal and inhalation, swallowing and aspiration, and introduction of the agent through wounds caused by injury and/or surgical intervention as mechanisms of NTM transmission.^(6)^

Population data from North America, Europe, and Australia show that the prevalence of NTM related to pulmonary disease continues to increase, and prevalence is lower in Europe than in North America and Australia. In regions of the United States, the prevalence in 2007 was 47 cases/100,000 population.^(7-9)^

According to their pathogenicity, NTM can be potentially or rarely pathogenic, or strictly environmental. Therefore, in order to evaluate the clinical significance of their isolation, the American Thoracic Society (ATS)^(10)^ has established clinical, radiological and bacteriological criteria to characterize non-tuberculous mycobacterial pulmonary disease (NTMPD). Potentially pathogenic NTM can cause various localized forms of disease that can affect the lungs, lymph nodes, skin, and joints, as well as take the disseminated form. When untreated, they can prove fatal or leave severe sequelae.^(11-13)^

Recent studies conducted in Central and South America, which are not multicenter, have contributed to the understanding of the epidemiology of NTMPD. The common limitation of all these studies is the lack of a reliable denominator in a defined population, making erroneous estimates regarding the frequency of NTMPD. Clinical diagnosis is made difficult because of the similarity of symptoms with other pulmonary conditions such as tuberculosis. Therefore, NTMPD requires a specific diagnosis in order to assertively define the therapeutic regimen.^(14)^

## OBJECTIVE

To present the frequency and species diversity of non-tuberculous mycobacteria, estimate the prevalence of non-tuberculous mycobacterial pulmonary disease, describe the epidemiological profile, and determine the follow-up of patients with non-tuberculous mycobacterial pulmonary disease living in a region with a high burden of tuberculosis.

## METHODS

This is a retrospective observational study, covering a cohort of patients with NTMPD from 2000 to 2009 and followed until December 2017, to evaluate the outcome of cases.

Patients of both genders, aged over 15 years, living in the municipalities of the Metropolitan Region of Baixada Santista, in the State of São Paulo, with a laboratory diagnosis of pulmonary disease by microbiological criteria according to the ATS confirmation criteria.^(10)^ This includes identification of the same species in two positive cultures obtained from separate sputum samples or one positive culture from bronchial lavage or aspirate, were considered cases of NTMPD.^(6,10,15)^

The definition of a confirmed case of NTMPD was established according to the ATS criteria^(10)^ and refers to every patient with two positive cultures from non-sterile material or one culture from sterile material from a patient submitted to investigation, and the case may have occurred more than once during the study period.

The study population consisted of patients who met the definition of confirmed case of NTMPD, living in one of the nine municipalities that make up the metropolitan region of Baixada Santista, between January 1, 2000 and December 31, 2009. In the selection of confirmed cases for epidemiological evaluation, according to the *Sistema de Vigilância Estadual do Estado de São Paulo* (TBWeb), when there was more than one notification in the study period, only the first was considered for analysis. All selected confirmed cases were followed up in TBWeb until December 31, 2017 for the analysis of treatment outcome (cure, abandonment, failure, death, and transfer). Patients under 15 years of age, who did not meet the ATS criteria or with missing information with TBWeb were excluded.

The Metropolitan Region of Baixada Santista was established by state complementary law 815, of July 19^th^ 1996 (https://www.al.sp.gov.br/repositorio/legislacao/lei.complementar/1996/lei.complementar-815-30.07.1996.html). It is composed of nine municipalities in the State of São Paulo (Santos, São Vicente, Guarujá, Bertioga, Praia Grande, Itanhaém, Cubatão, Mongaguá and Peruíbe), totaling 1,476,820 inhabitants, being the third region of the state in population.

Data were collected from the databases of the *Instituto Adolfo Lutz* (IAL) in Santos to obtain laboratory data, and from the *Programa Estadual de Controle da Tuberculose da Divisão de Tuberculose do Centro de Vigilância Epidemiológica do Estado de São Paulo* to obtain clinical epidemiological data (TBWeb Information System). Demographic data were obtained from the database of the *Instituto Brasileiro de Geografia e Estatística* (IBGE).

The variables of interest referred to sociodemographic characteristics (gender, date of birth, and municipality of residence), to the current and past history of pulmonary tuberculosis (PTB; if PTB in the past, and type of discharge from previous PTB), to the confirmation criteria, to treatment-related aspects (supervised treatment, hospitalization for tuberculosis (TB), change of regimen, regimens used, and termination of current treatment), and to comorbidities and conditions (HIV/AIDS, among others). Variables of interest in which no information (no information - SI) was observed in the notification form were kept in the analysis to allow discussion of the cases.

The culture laboratory tests were performed by IAL - Santos and the identification tests by IAL - Central. Culture was performed in Middlebrook 7H9 medium of the MGIT 960 system and in Löwenstein-Jensen medium, after sputum digestion and decontamination by the Petroff method.^(16)^ Identification of the isolates was performed by the PRA-hsp65 method and some phenotypic identification tests.^(17)^

The database used in this study was initially formed with the laboratory information obtained from the registration book, and patients who met the case definition and inclusion criteria were selected. Subsequently, these data were compared with the TBWeb database, which is used by the epidemiological surveillance for monitoring PTB in the state of São Paulo. This search aimed to verify the variables of interest to the study that were not included in the laboratory data.

Finally, duplicates were eliminated by pairing the name, date of birth, patient’s address, and mother’s name. Then, consistency analysis and analysis of the results were performed with SPSS software, version 20.

The analysis of frequency and diversity of NTM species was expressed as mean or proportion. The distribution of confirmed NTMPD cases was made according to time and space, followed by a descriptive analysis of the main characteristics of interest of the cases. For comparative analyses between proportions, Pearson’s χ^2^ test and Fisher’s exact test were used, and for continuous variables, the Kruskal-Wallis test.

For the estimation of the annual prevalence of isolates, the number of isolates found in the period was taken as the numerator, and for the estimation of confirmed NTMPD cases in the period of interest, the numerator was the total number of confirmed cases. For both situations, the denominator used was the average population aged 15 years or older in the period.

The research project was approved by the IAL Ethics Committee (136487/2010). Since this is a retrospective cohort study and it was impossible to obtain the consent form, we are responsible and/or committed to the privacy and confidentiality of data used in the project, fully preserving the anonymity of patients, according to the recommendations of resolution 196 of October 10, 1996 of the National Health Council for scientific research on human subjects.

## RESULTS

### Isolated non-tuberculous mycobacterial species

From 2000 to 2009, 319 NTM isolates were identified. Of these, 10.0% (32/319) could not be identified to species level due to technical limitations, and Runyon’s classification was used.^(18)^ In 2.2% (7/319) of isolates, the presence of *Mycobacterium tuberculosis* and one NTM species was detected concomitantly (mixed culture), and in 4.4% (14/319), a mixed culture of NTM species was detected (Table 1).

**Table 1:** Isolation frequency of non-tuberculous mycobacteria

Identification	Isolated n (%)
*M. kansasii*	91 (28.5)
*M. fortuitum*	53 (16.6)
MAC	37 (11.6)
*Mycobacterium spp.*	30 (9.4)
CLA	14 (4.4)
CRA	11 (3.4)
*M. gordonae*	10 (3.1)
*M. intracellulare/M. chimaera*	9 (2.8)
*M. abscessus*	8 (2.5)
*M. peregrinum*	8 (2.5)
*M. nonchromogenicum*	4 (1.3)
CLE	3 (0.9)
CLF	3 (0.9)
*M. flavescens*	3 (0.9)
*M. scrofulaceum*	3 (0.9)
*M. tuberculosis/M. kansasii*	3 (0.9)
*M. immunogenum*	2 (0.6)
*M. intracellulare*	2 (0.6)
*M. lentiflavum*	2 (0.6)
*M. szulgai*	2 (0.6)
*M. terrae*	2 (0.6)
*M. tuberculosis /MNT*	2 (0.6)
*M. terrae/M. triviale*	2 (0.6)
*M. fortuitum/ M. chelonae*	2 (0.6)
CRE	1 (0.3)
*M. asiaticum*	1 (0.3)
*M. bohemicum*	1 (0.3)
*M. celatum*	1 (0.3)
*M. chelonae*	1 (0.3)
*M. mucogenicum*	1 (0.3)
*M. monacense*	1 (0.3)
*M. mucogenicum*	1 (0.3)
*M. holsaticum*	1 (0.3)
*M. shimoidei*	1 (0.3)
*M. tuberculosis/ M. intracellulare*	1 (0.3)
*M. tuberculosis/ M. gordonae*	1 (0.3)
*M. abscessus/ M. peregrinum*	1 (0.3)
Total	319 (100.0)

MAC: *Mycobacterium avium complex*; CLA: *Mycobacterium acromogenous slow-growing*; CRA: *Mycobacterium acromogenous fast-growing*; CLE: *Mycobacterium scotochromogenous slow-growing*; CLF: *Mycobacterium photochromogenous slow-growing*; MNT: non-tuberculous mycobacteria; CRE: *Mycobacterium scotochromogenous fast-growing*.

For 83.4% (266/319) isolates, species identification was obtained, with *Mycobacterium kansasii* being the species with the highest number of isolates (34.2%; 91/266), followed by *Mycobacterium fortuitum* (19.9%; 53/266) and *Mycobacterium avium* complex (MAC) (13.9%; 37/266). In 11.3% (30/266) of the isolates, identification could not be obtained and were considered only as *Mycobacterium spp*. due to the absence of the molecular profile in the algorithm used for interpretation of the PRA-hsp65 technique.

During the study period, the highest number of isolations occurred in 2009 (16.6%; 53/319) with a higher frequency of the *Mycobacterium fortuitum* species (4.7%; 15/319) (Table 2).

**Table 2:** Annual distribution of the frequency of non-tuberculous mycobacteria species, by number of isolates, from 2000 to 2009. Metropolitan region of Baixada Santista, São Paulo, SP, Brazil

Species	Isolated	n (%)	2000	2001	2002	2003	2004	2005	2006	2007	2008	2009
CLA	14	(4.4)	2	3	2			1	2		1	3
CRA	11	(3.4)			1			1	1		2	6
CLE	3	(0.9)	1					2				
CLF	3	(0.9)									3	
CRE	1	(0.3)			1							
*M. kansasii*	91	(28.5)	5	4	13	14	10	13	13	4	8	7
*M. fortuitum*	53	(16.6)	3	3	4	6	3	8	4	2	5	15
MAC	37	(11.6)		2	9	2	4	3	7	4	2	4
*Mycobacterium spp.*	30	(9.4)				16	14					
*M. gordonae*	10	(3.1)			1				4	2		3
*M. abscessus*	8	(2.5)				1		3	2	1		1
*M. peregrinum*	8	(2.5)		1	2		3			1		1
*M. nonchromogenicum*	4	(1.3)		1	1				1	1		
*M. flavenscens*	3	(0.9)					1		2			
*M. scrofulaceum*	3	(0.9)							1	2		
*M. immunogenum*	2	(0.6)							1			1
*M. intracellulare*	2	(0.6)						1	1			
*M. lentiflavum*	2	(0.6)						1			1	
*M. mucogenicum*	2	(0.6)							1		1	
*M. szulgai*	2	(0.6)								1		1
*M. terrae*	2	(0.6)		2								
*M. asiaticum*	1	(0.3)							1			
*M. bohemicum*	1	(0.3)						1				
*M. celatum*	1	(0.3)										1
*M. chelonae*	1	(0.3)						1				
*M. monacense*	1	(0.3)									1	
*M. holsaticum*	1	(0.3)										1
*M. shimoidei*	1	(0.3)			1							
Mixed culture	21	(6.6)	0	2	4	1	1		1	1	2	9
Total	319	(100)	11 (3.4)	18 (5.6)	39 (12.2)	40 (12.5)	36 (11.3)	35 (11.0)	42 (13.2)	19 (6.0)	26 (8.2)	53 (16.6)

CLA: achromogenic slow-growing mycobacteria; CLE: scotochromogenic slow-growing mycobacteria; CLF: photochromogenic slow-growing mycobacteria; CRA: achromogenic fast-growing mycobacteria; CRE: scotochromogenic fast-growing mycobacteria; MAC: *Mycobacterium avium complex*.

### Identification of patients (clinical, epidemiological and sociodemographic profile)

**Figure 1:**
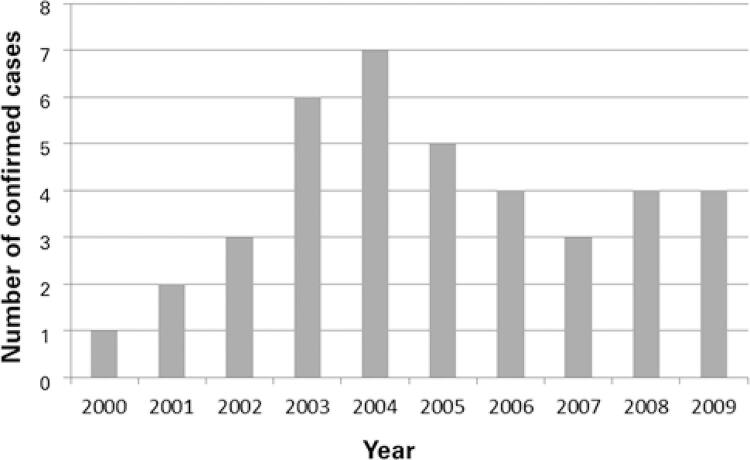
Distribution of patients according to confirmed cases of pulmonary disease caused by non-tuberculous mycobacteria

The 319 NTM isolates were from 257 patients submitted to investigation for pulmonary infection. Of these, 15.2% (39/257) were identified as confirmed cases, and 2004 was the year with the highest number of cases (7 cases/year) (Figure 1). For the epidemiological analysis of the confirmed cases, according to the established selection criteria, 61.5% (24/39) of the cases were selected in the TBWeb system and analyzed. The most frequent species among the reported cases were *Mycobacterium kansasii* (37.5%; 9/24) and *Mycobacterium fortuitum* (12.5%; 3/24). The remaining 15 cases could not be analyzed in TBWeb due to the lack of information/report.

Analyzing the sociodemographic characteristics of these cases, 79.2% (19/24) were men, and 41.7% (10/24) were over 50 years old, with a mean of 44.0 years of age. Among the comorbidities evaluated, 37.5% (9/24) had positive serology for HIV, and for those for which we had information, 15.4% (2/13) had diabetes. There was no record of other comorbidities; 45.8% (11/24) had a past history of PTB (Table 3).

**Table 3:** Characteristics of patients with confirmed cases of non-tuberculous mycobacteria infection, reported according to isolated species

Characteristics	*Mycobacterium kansasii* n=9 n (%)	*Mycobacterium fortuitum* n=3 n (%)	MAC* n=5 n (%)	Other species n=7 n (%)	Total n=24 n (%)
Sex					
Women	1 (11.1)	1 (33.3)	2 (40.0)	1 (14.3)	5 (20.8)
Men	8 (88.9)	2 (66.7)	3 (60.0)	6 (85.7)	19 (79.2)
Age group, years					
15-25	0	0	1 (20.0)	1 (14.3)	2 (8.3)
26-30	0	1 (33.3)	0	1 (14.3)	2 (8.3)
31-35	1 (11.1)	0	1 (20.0)	1 (14.3)	3 (12.5)
236-40	2 (22.2)	0	1 (20.0)	0	3 (12.5)
41-45	1 (11.1)	0	0	0	1 (4.2)
46-50	1 (11.1)	1 (33.3)	1 (20.0)	0	3 (12.6)
50 or more	4 (44.4)	1 (33.3)	1 (20.0)	4 (57.1)	10 (41.7)
HIV infection					
No	8 (88.9)	2 (66.7)	1 (20.0)	4 (57.1)	15 (62.5)
Yes	1 (11.1)	1 (33.3)	4 (80.0)	3 (42.9)	9 (37.5)
Diabetes					
No	5 (55.6)	1 (33.3)	2 (40.0)	3 (42.9)	11 (458.)
Yes	1 (11.1)	1 (33.3)	0	0	2 (8.3)
SI	3 (33.3)	1 (33.3)	3 (60.0)	4 (57.1)	11 (45.8)
Past TB*					
No	4 (44.4)	1 (33.3)	4 (80.0)	3 (50.0)	12 (52.2)
Yes	5 (55.6)	2 (66.7)	1 (20.0)	3 (50.0)	11 (47.8)
Outcome past TB					
Cure	3 (33.3)	1 (33.3)	1 (20.0)	2 (28.6)	7 (29.2)
Abandonment	0	0	0	0	0
Failure	0	0	0	0	0
SI	6 (66.7)	2 (66.7)	4 (80.0)	5 (71.4)	17 (70.8)
Supervised treatment					
Yes	6 (22.2)	1 (33.3)	4 (80.0)	2 (28.6)	13 (37.5)
No	2 (66.7)	1 (33.3)	0	2 (28.6)	5 (37.5)
SI	1 (11.1)	1 (33.3)	1 (20.0)	3 (42.9)	6 (25.0)
Treatment regimen					
RHZ	4 (50.0)	1 (50.0)	4 (80.0)	3 (75.0)	12 (63.2)
2RHZE	3 (37.5)	1 (50.0)	1 (20.0)	0	5 (26.3)
Other drugs	1 (12.5)	0	0	1 (33.3)	2 (10.5)
Current outcome					
Cure	3 (33.3)	2 (66.7)	2 (40.0)	4 (57.1)	11 (45.8)
Dropout	0	0	0	0	0
Death TB	0	0	0	0	0
TB/HIV death	0	0	1 (20.0)	0	1 (4.2)
Failure	0	0	0	0	0
Change of diagnosis	6 (66.7)	1 (33.3)	1 (20.0)	1 (14.3)	9 (37.5)
Transfer	0	0	1 (20.0)	1 (14.3)	2 (8.3)
SI	0	0	0	1 (14.3)	1 (4.2)

*When the sum of the categories of each variable was smaller than the number of cases studied, the difference was due to the absence of information.

MAC: *Mycobacterium avium complex*; TB: tuberculosis; SI: no information; RHZ: rifampicin, isoniazid and pyrazinamide; RHZE: rifampicin, isoniazid, pyrazinamide and ethambutol.

**Figure 2:**
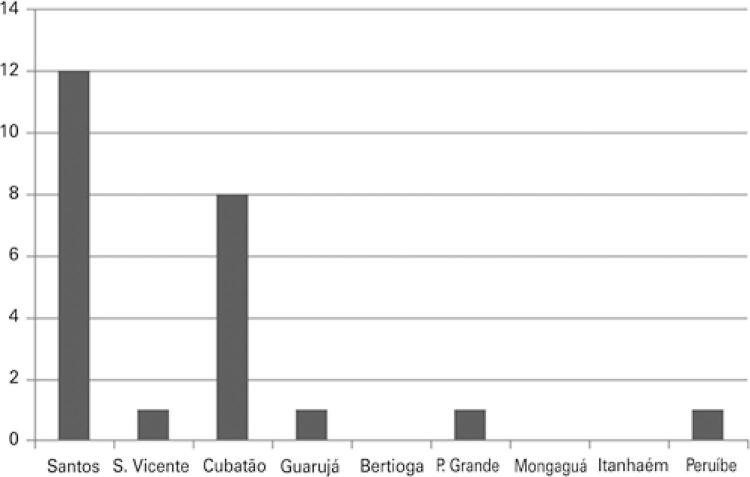
Distribution of patients confirmed with pulmonary disease due to non-tuberculous mycobacteria

According to the municipality of residence, the highest occurrence of patients was observed in the municipalities of Santos and Cubatão, with 50.0% (12/24) and 33.3% (8/24), respectively (Figure 2).

Information about the X-ray examination was available for 66.7% (16/24) of the patients. Of these, 25.0% (4/16) had X-ray with lung cavity and 68.8% (11/16) with suspected PTB.

### Treatment outcome

Information regarding current TB treatment was obtained for 79.2% (19/24) of the cases. Of these, 63.1% (12/19) used the basic regimen (isoniazid, rifampicin, and pyrazinamide - the old basic treatment regimen for new cases), and 54.12% (13/24) used supervised treatment.

In the analysis of the outcome of the current treatment, 45.8% (11/24) were terminated as a cure, and 37.5% (9/24) had a change of diagnosis, *i.e*., patients notified with tuberculosis and who, in the course of the investigation, were detected to have a mycobacteriosis. For 8.3% (2/24), there was a transfer of care unit, and 4.2% (1/24) died from another cause. Only one of the cases reported on TBWeb did not show closure by the final follow-up date (December 31, 2017) (Table 3). Cure as closure among the 11 patients who had PTB in the past was 63.6% (7/11).

Table 4 shows the annual prevalence of isolates and confirmed cases of NTMPD over the study period, with the highest prevalence of isolates in 2009.

**Table 4:** Annual prevalence of isolates and cases of pulmonary disease due to non-tuberculous mycobacteria*

Year	Prevalence of isolates*	Prevalence of confirmed cases*
2000	0.9 (n=10)	0.1 (n=1)
2001	1.5 (n=16)	0.2 (n=2)
2002	3.3 (n=34)	0.3 (n=3)
2003	3.4 (n=32)	0.5 (n=6)
2004	3.0 (n=26)	0.5 (n=7)
2005	2.9 (n=25)	0.3 (n=5)
2006	3.5 (n=35)	0.4 (n=4)
2007	1.6 (n=14)	0.3 (n=3)
2008	2.2 (n=20)	0.3 (n=4)
2009	4.4 (n=45)	0.3 (n=4)
Total	n=257	n=39

*Prevalence per 100 thousand inhabitants. The total number of confirmed cases in the year was divided by the total average resident population in Baixada Santista during the period studied, according to data from the IBGE.

## DISCUSSION

Currently, there are few articles referring to the epidemiology of confirmed cases of NTMPD, especially those relating it to factors such as abandonment of a PTB treatment in the past and diseases other than AIDS/HIV.^(19)^ Marques et al.,^(20)^ in the period from 2011 to 2014, evaluated 1,014 patients with pulmonary NTM isolation with respect to meeting the ATS microbiological criteria and found 44.2% of patients with NTPMD, mainly caused by *Mycobacterium kansasii, Mycobacterium abscessus, Mycobacterium intracellulare, Mycobacterium avium* and *Mycobacterium szulgai*.

Another difficulty is meeting the criteria established by the ATS for the confirmation of NTMPD. One study confirmed 56% of patients with NTM isolation in 2005 and 2006.^(21)^In the Baixada Santista region,^(22)^between 2000 and 2005, this rate was lower (19.2%). In the present study, of the 257 cases identified, 39 were confirmed cases and 24 of these were analyzed for epidemiological characteristics on TBWeb. These data highlight the need for improvement in the diagnosis of NTMPD, in the intensification of efforts to comply with the criteria established by the AST, and in the flow of case referrals, since the confirmatory diagnosis and treatment follow-up in the region are centralized in the reference laboratory/ambulatory of the region.

There are still few studies on the prevalence of NTM. In the literature, we found a prevalence of 41.3/100,000 population,^(14)^7.2/100,000 population,^(21)^ and 10.0/100,000 population^(23)^ - even higher than the prevalence found in this study (2.09/100,000 population).

Studies in Ontario, Canada,^(24)^and Taiwan^(25)^ showed that in the same period of this study there was an increase in the incidence of NTMPD. In our study, no significant differences were observed in the number of confirmed cases over the period, which may strengthen the hypothesis of underestimated numbers, since there is no mandatory reporting of NTMPD cases in our country.

Research outside Brazil has shown that species of the MAC are the most common NTM among isolates^(18,21)^ and confirmed cases.^(19,26)^A study in Colombia showed that in HIV-positive patients, *M. avium* was the most isolated *mycobacterium* (4.2%), even ahead of *M. tuberculosis* (1.2%), and no other species were isolated.^(3)^

The distribution of NTMs is different in the various regions of the world, and possibly the lack of multicenter studies in the literature makes it difficult to understand this diversity in research results. Studies show that, in industrialized countries, the most prevalent species are MAC and *M. kansasii*.^(14)^ In the last two reports published in the United States on the prevalence of NTM, *M. avium* was the most isolated species, followed by *M. fortuitum* and *M. kansasii.* Brazil shows diversity in the prevalence of NTM species that cause pulmonary disease. In a study conducted in 2012, *Mycobacterium massiliense* and the *Mycobacterium simiae* complex were dominant.^(27)^ In the region analyzed in the present study, *M. kansasii* had the highest occurrence (28.5%), followed by *M. fortuitum* (16.6%). The work performed by the NTM-NET network evaluated data from 62 laboratories in 30 countries distributed over six continents. This study showed important geographic differences in species distribution and that these differences determine the type of NTMPD in each location.^(8)^

In our study, the main species in Baixada Santista were *M. kansasii*, followed by *M. fortuitum* and MAC, among the isolates.

The identification of NTM species is still very centralized in reference laboratories, since there is no rapid and unique methodology that allows the identification of all species currently described. Only the sequencing of a few genes allows this identification. However, the routine methodology used in this study, PRA-hsp65, has limitations. Many profiles are not yet described in the algorithm used for interpretation, and therefore isolates were characterized only by Runyon classification. Other isolates, which did not grow well and presented an undescribed profile, were identified only at the genus level.

Among the reported cases, the most frequent species was *M. kansasii*, in 37.5% (9/24).

Non-tuberculous mycobacteria disease has been of increasing interest in the literature, possibly because of the increased incidence of NTM infections in patients with HIV infection. Recently, studies have been frequent in non-HIV-infected patients with *Mycobacterium intracellulare* (59.1%) and *M. avium* (14.3%) species as the most isolated.^(28)^

It is important to highlight that, among the potentially pathogenic species, those belonging to the *M. avium* and *M. kansasii* complex (slow-growing mycobacteria) are the most commonly isolated, and both cause mainly pulmonary infection, the former being frequently responsible for disseminated disease and death in HIV-positive patients.^(18,23,29)^By analyzing only patients with confirmed cases of NTMPD, our results showed that, if we evaluate the data independently of HIV serology, *M. kansasii* still remains the most commonly isolated species (37.5%), followed by MAC (20.8%) and *M. fortuitum* (12.5%). *M. kansasii* still remained the most isolated species (37.5%), followed by MAC (20.8%) and *M. fortuitum* (12.5%) - results similar to those found in another study.^(29)^Mycobacterium *avium* complex was more frequent, taking into account only isolations from HIV-positive patients (44.0%), but considering HIV-negative patients, *M. kansasii* (53.3%) stands out as the most isolated species, as also observed in a study conducted in the same region^(19)^ in the period from 2000 to 2005, confirming that the profile of NTM disease remains the same.

Studies show HIV infection as a comorbidity associated with NTMD, which corroborates the data observed in our study (37.5%). It is also noted that individuals older than 50 years and those with positive HIV serology constitute a risk group. A study conducted in the state of Rio Grande do Sul, an area of high HIV incidence in Brazil, describes that 58% of HIV patients were confirmed with NTMPD.^(29)^

A study in Taiwan, at a pulmonary rehabilitation center in Kuwait, showed that 55.5% of the patients with NTMPD were 65 years of age or older.^(25)^In a North American study, the mean age of patients with NTMPD was 55.8 years.^(30)^These results differ from the mean observed in the present study (44 years), but both agree that most affected individuals are over 50 years of age.

The literature shows a different distribution of the NTMPD according to gender. Two other studies^(21,30)^ reported that 95.0% and 57.7% of the patients were female, data that corroborate with other authors.^(25,27)^However, in a study by Zamarioli et al.,^(22)^ The latter result, found in the same population as in the present study, shows that the prevalence of the female gender is higher, considering the current rate of 20.8% and pointing out that women are less affected in our region. These data suggest that women in our country are increasingly attentive to preventive care of diseases and, consequently, become less vulnerable to the involvement of NTMPD. Even so, we can consider the performance of control programs in the search for diagnosis of the disease in the various detention centers in the Baixada Santista region, revealing a higher number of males.

In the present study, information on past PTB was observed in 45.8% of the patients with confirmed cases, numbers close to those reported in a study conducted in Kuwait (42.6%) and also observed by another Brazilian study,^(29)^in which this is pointed out as the principal risk factor associated with NTMPD (76%).^(27,31)^As oriented by the Ministry of Health, the non-recording of information on tuberculosis in the past should be done only when the possibilities of investigating the patient’s previous history have been exhausted.^(32)^The disease is present in most patients, considering the X-ray results with pulmonary cavity (16.7%) or with suspected PTB (45.8%).

In Africa, a study involving ten hospitals in western Kenya between 2007 and 2010 highlighted that, among the 361 PTB patients, NTMs were present in 4.2% of cases. Studies conducted in the region of interest suggest that previously diagnosed multidrug resistant PTB patients may have NTM-related pulmonary disease.^(14)^

Records regarding NTMPD are still scarce, since diseases caused by NTM are not compulsorily notifiable. A large number of cases do not receive laboratory confirmation, and this fact may reflect both a failure in the health care systems and a lack of patient compliance with correct medical follow-up. According to TBWeb, in our study, 45.8% of the notified patients were cured, and 37.5% had a change of diagnosis. The change of diagnosis is signaled when a change in diagnosis occurs and it is elucidated that it was not a case of TB. Thus, greater attention to cases of mycobacteriosis is necessary, especially to differentiate them from cases of PTB, since NTM species are naturally resistant to the antibiotics used in the treatment of TB, and a change in treatment is essential for case improvement. New population-based epidemiological studies may help in the knowledge and mapping of NTM, favoring the treatment and follow-up of patients.

The present study was limited by the use of secondary data, which implied the observation of incomplete or incorrect data in the notification form and the underreporting of TB cases.

## CONCLUSION

The Baixada Santista region has a different profile of non-tuberculous mycobacterial pulmonary disease due to the higher frequency of isolation of *M. kansasii, M. fortuitum* and *M. avium complex*. Despite the effort to identify these species, these patients remain under inadequate treatment, since no change in diagnosis occurs, as observed in the notification system. Therefore, the detection of a non-tuberculous *mycobacterium* should generate a closer evaluation of the case.

Since non-tuberculous mycobacteria species have different levels of pathogenicity, consideration should be given to analyzing prevalence as a function of species. This information is important for establishing a non-tuberculous mycobacteria surveillance program, considering only those species that actually cause disease in the population of a given region - an action that will be more specific, but even more effective.
